# Supplementary material for: Plasmonic and Photothermal Effects of CuS Nanoparticles Biosynthesized from Acid Mine Drainage with Potential Drug Delivery Applications
Source: Int J Mol Sci. 2023 Nov 18;24(22):16489. doi: 10.3390/ijms242216489 (PMC10671710; doi:10.3390/ijms242216489)
Supplement: Supplementary file 1 [file ijms-24-16489-s001.zip › ijms-2663695-supplementary.pdf]

International Journal of
Molecular Sciences

MDPI

Supplementary Material

Plasmonic and Photothermal Effects of CuS Nanoparticles Biosynthesized from Acid Mine Drainage with Potential Drug Delivery Applications

Hernán Escobar-Sánchez [1], Claudio Carril Pardo [2], Noelia Benito [1], Jacobo Hernández-Montelongo [3], Iván Nancucheo [4] and Gonzalo Recio-Sánchez [4,*]

[1] Departamento de Física, Universidad de Concepción, Concepción 4070386, Chile; hescobar2016@udec.cl (H.E.-S.); noelia.benito@udec.cl (N.B.)
[2] Facultad de Ciencias de la Salud, Universidad San Sebastián, Concepción 4080871, Chile; claudio.carril@uss.cl (C.C.P.)
[3] Departamento de Ciencias Matemáticas y Físicas, Universidad Católica de Temuco, Temuco 4823302, Chile; jacobo.hernandez@uct.cl (J.H.M)
[4] Facultad de Ingeniería, Arquitectura y Diseño, Universidad San Sebastián, Concepción 4080871, Chile; ivan.nancucheo@uss.cl (I.N.); gonzalo.recio@uss.cl (G.R.S.)
* Correspondence: author: gonzalo.recio@uss.cl

Figure S1 show the molecular structure of the caffeic acid which is characterized by a by a phenylpropanoid (C6-C3) structure with a 3,4-dihydroxylated aromatic ring joined to a carboxylic acid.

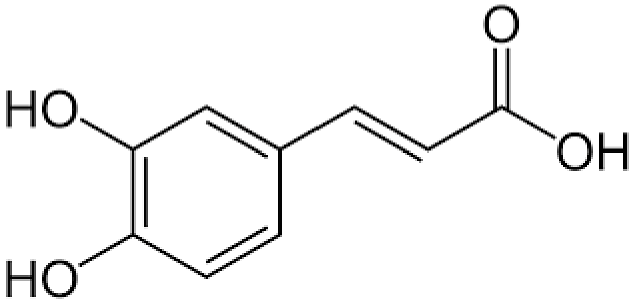

Figure S1. Molecular structure of caffeic acid.

In order to study the interaction between the CuS nanoparticles and caffeic acid, FTIR measurements were performed. Figure S2 shows the FTIR spectrum of CuS nanoparticles which did not present any absorption peaks. FTIR spectrum of caffeic acid showed several intense absorption bands. According to the literature, the bands observed between 4000 and 2500 cm^{-1} can be related to the OH stretching vibrations and CH stretching modes of acyclic chain and benzene moiety [73,74]. The absorption peaks located at 1648, 1450 and 1280 cm^{-1} can be assigned to C=O, C-C and C-H stretching vibration, respectively [75]. Moreover, the peaks observed between 1120 and 800 cm^{-1} can be correlated to C-C-C bending modes of the aromatic ring and the bending modes of the carbonyl group [75].

To get deeper knowledge abut the interaction of CuS nanoparticles and caffeic acid, 5 mg of CuS nanoparticles were incubated into a saturated aqueous solution of caffeic acid (1 mM) during 24 hours. After that, the CuS nanoparticles were recovered by centrifugation and dry at room temperature. The FTIR spectrum (CuS+caffeic acid) is also shown in Figure 2S. The spectrum was charaterizd by a wide absorption band between 3500 and 2500 cm^{-1} of wavenumber. However, any peaks could be related to the caffeic acid molecular structure.

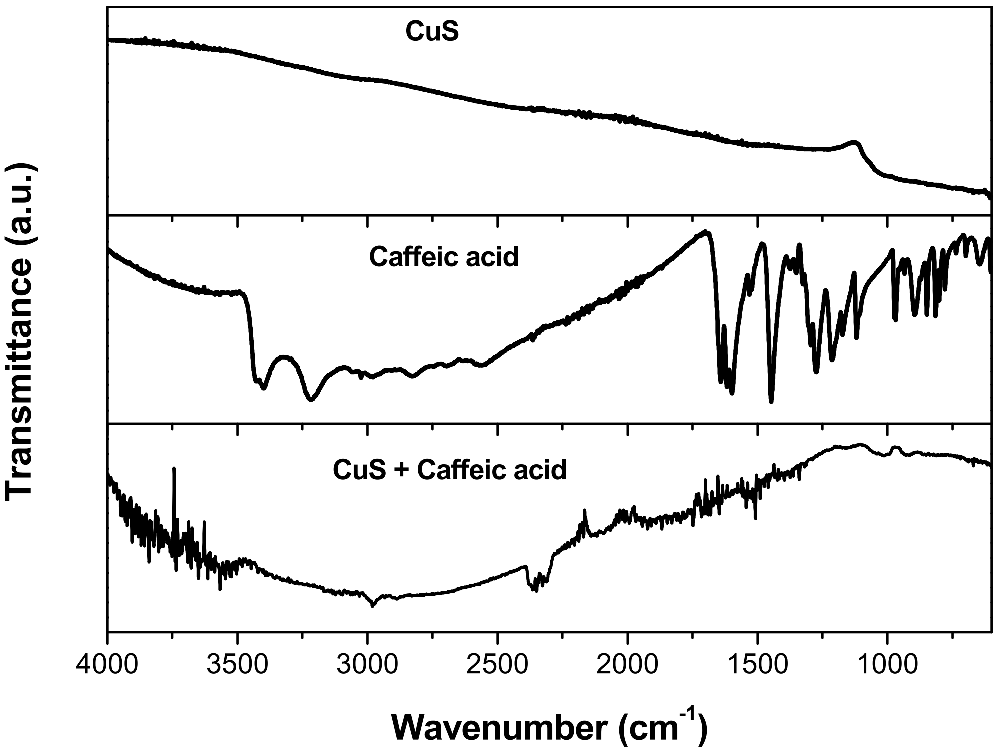

Figure S2. FTIR spectra of CuS nanoparticles, caffeic acid and CuS incubated into an aqueous solution of caffeic acid.

References

73. Świsłocka, R. Spectroscopic (FT-IR, FT-Raman, UV Absorption, ^{1}H and ^{13}C NMR) and Theoretical (in B3LYP/6-311++G** Level) Studies on Alkali Metal Salts of Caffeic Acid. Spectrochim. Acta A Mol. Biomol. Spectrosc. **2013**, *100*, 21–30. https://doi.org/10.1016/j.saa.2012.01.048.
74. Tosovic, J. Spectroscopic Features of Caffeic Acid: Theoretical Study. Kragujevac Journal of Science **2017**, 99–108. https://doi.org/10.5937/KgJSci1739099T.
75. Catauro, M.; Barrino, F.; Dal Poggetto, G.; Crescente, G.; Piccolella, S.; Pacifico, S. New SiO_2/Caffeic Acid Hybrid Materials: Synthesis, Spectroscopic Characterization, and Bioactivity. Materials **2020**, *13*, 394. https://doi.org/10.3390/ma13020394.
